# Bevacizumab combined with chemotherapy for ovarian cancer: an updated systematic review and meta-analysis of randomized controlled trials

**DOI:** 10.18632/oncotarget.12926

**Published:** 2016-10-26

**Authors:** Yu Shen Wu, Lin Shui, Dan Shen, Xiaopin Chen

**Affiliations:** ^1^ Department of Oncology, The First Affiliated Hospital of Chongqing Medical University, Chongqing, P.R. China

**Keywords:** bevacizumab, ovarian cancer, meta-analysis, survival, adverse event

## Abstract

**Background:**

This meta-analysis was updated with results from a new trial and final data to reassess the efficacy and safety of bevacizumab combined with chemotherapy in ovarian cancer (OC).

**Methods:**

Randomized controlled trials (RCTs) were searched in PubMed, EMBASE, Cochrane clinical trials, Web of Science and clinicaltrial.gov databases. Outcomes included the progression-free survival (PFS), overall survival (OS), objective response rate (ORR) and common adverse events. The hazard ratio (HR), risk ratio (RR) and odds ratio (OR) were pooled when the meta-analysis was performed.

**Results:**

Five RCTs with 4994 patients were included. In overall newly diagnosed OC, bevacizumab combined with chemotherapy did not significantly improve PFS (HR 0.85, 95%CI 0.70-1.02) or OS (HR 0.94, 95%CI 0.84-1.05). In the high-risk progression subgroup, the addition of bevacizumab significantly improved PFS (HR 0.76, 95%CI 0.68-0.84) and OS (HR 0.85, 95%CI 0.74-0.96). In recurrent OC, the addition of bevacizumab to chemotherapy significantly extended PFS (HR 0.53, 95%CI 0.45-0.63) and OS (HR 0.87, 95%CI 0.77-0.99). The ORR was improved (OR 2.37, 95%CI 1.99-2.82) in the overall population. Bevacizumab increased the incidence of hypertension (RR 21.27, 95%CI 9.42-48.02), proteinuria (RR 4.77, 95%CI 2.15-10.61), bleeding (RR 3.16, 95%CI 1.59-6.30), GI perforations (RR 2.76, 95%CI 1.51-5.03), arterial thrombosis events (RR 2.39, 95%CI 1.39-4.10) and venous thrombosis events (RR 1.43, 95%CI 1.04-1.96).

**Conclusions:**

Bevacizumab combined with chemotherapy significantly improved PFS and OS in both patients with high-risk of progression and patients with recurrent OC, with an increased incidence of common adverse events. However, no statistically significant survival benefit was identified in the front-line settings.

## INTRODUCTION

Ovarian cancer (OC) carries one of the worst prognoses among gynecological tumors and is the fifth cause of cancer death among women [1]. The standard treatments for ovarian cancer include appropriate surgery or cytoreduction followed by adjuvant chemotherapy in most patients. Evidence indicates that overall survival (OS) is increased in patients who receive systemic chemotherapy. However, 70% patients with advanced ovarian cancer will relapse and even die after the adoption of surgery and chemotherapy [2]. More novel therapeutic options are explored in the recent study.

Bevacizumab is an anti-VEGF antibody that has demonstrated activity in ovarian cancer [3]. Several randomized controlled trials (RCTs) have been launched to evaluate the efficacy and safety of bevacizumab combined with chemotherapy in ovarian cancer. However, the survival benefit of bevacizumab was different in these trials. In 2015, final data from the ICON7 trial indicated that progression-free survival (PFS) was not statistically improved [4], and this result differed from other trials [5–8]. In addition, an exploratory analysis from the ICON7 trial presented the survival benefit in high-risk patients as significantly improved [9], but this finding was inconsistent with the GOG-218 trial [8] that recruited a similar population. Notably, only the GOG-213 trial revealed that median OS was significantly extended in patients who received bevacizumab combined with chemotherapy treatment [5].

Furthermore, previously reported preliminary data could not accurately reflect the survival benefit. The most recently published meta-analysis indicated that OS was improved by bevacizumab combined with chemotherapy for newly diagnosed OC [10], whereas the final data from several trials did not support this opinion. In this present study, the final data and a new RCT (GOG-213) were included to reassess the efficacy and safety of bevacizumab combined with chemotherapy in ovarian cancer.

## RESULTS

### Included studies and study quality

**Figure 1:**
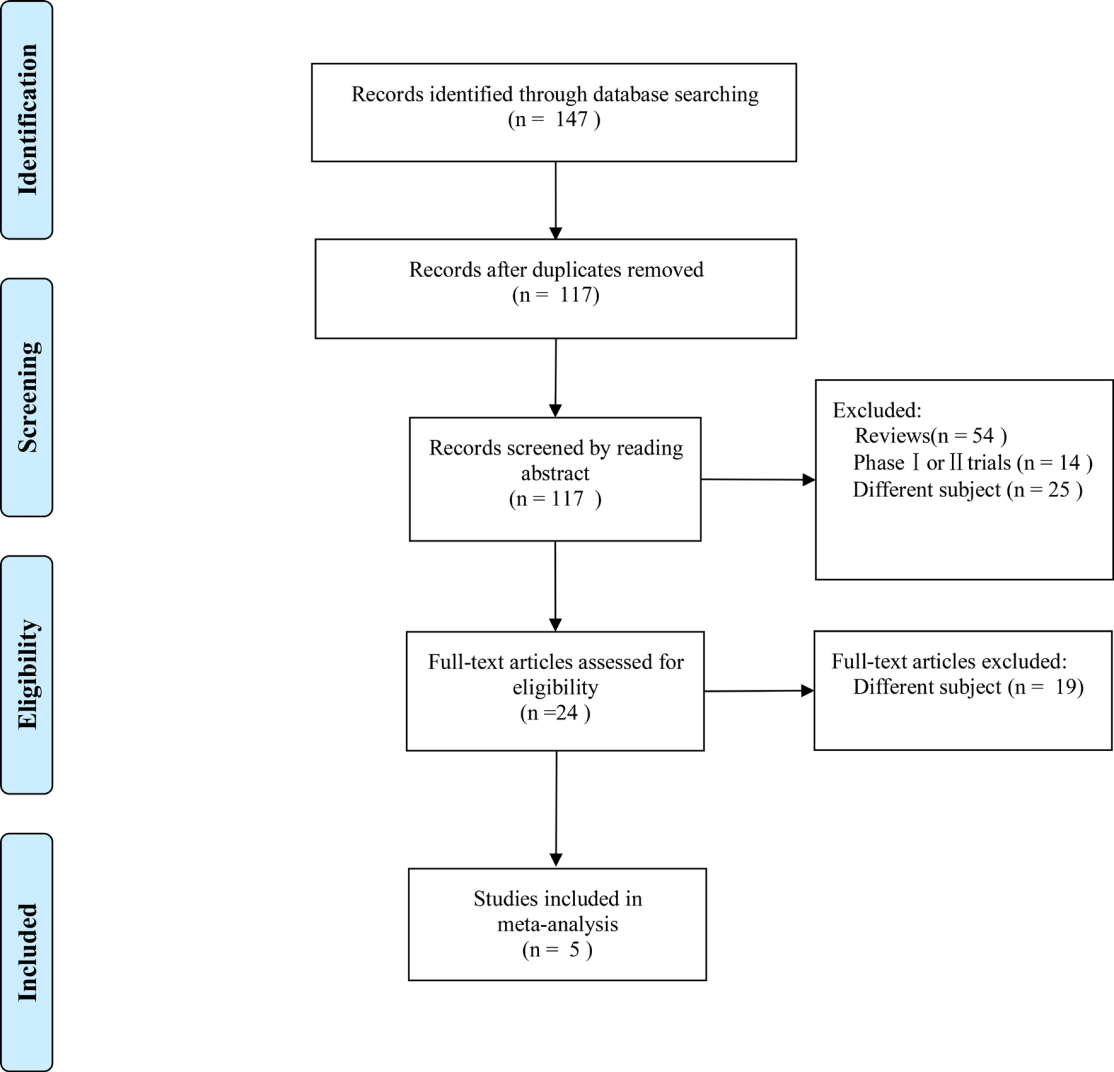
The process of study selection

**Figure 2:**
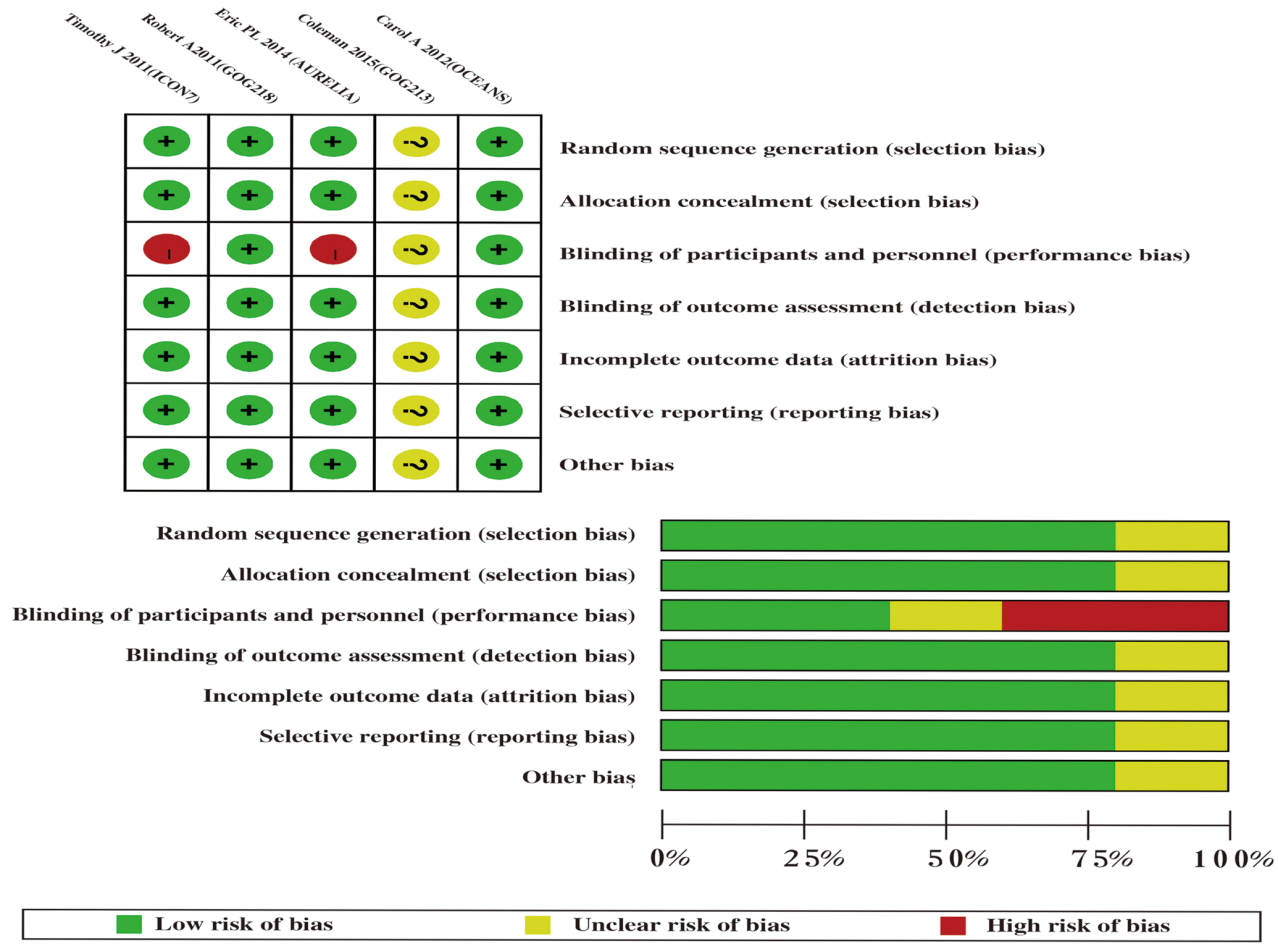
Risk of bias summary

We initially identified 147 articles from all searched databases, and 24 articles were retained after a full-text screening for inclusion in our review after excluding duplicates, reviews, and phase I and phase II trials. Finally, 5 randomized controlled trials with 4994 patients met our inclusion criteria (Figure 1). The main characteristics of 5 RCTs were summarized in Table 1, and the data of outcomes were summarized in Table 2. The overall risk of bias was judged to be low (Figure 2). The blinding of participants minimally influenced the survival time. The risk of bias was unclear in the study that was published in an abstract form.

**Table 1:** Characteristics of 5 RCTs

	GOG218	ICON7	OCEANS	AURELIA	GOG213
Primary endpoint	PFS	PFS	PFS	PFS	OS
Patients enrolled	Stage III (incompletely resectable) or stage IV	Stage I-III or StageIV or Inoperable Stage III	Platinum-sensitive recurrent ovarian cancer (recurrence ≥6 months after completing platinum-based therapy)	Platinum-resistant recurrent ovarian cancer that had progressed ≤6 month after completing platinum-based therapy	Platinum-sensitive recurrent ovarian cancer
GOC/ECOG PS	GOG PS 0-2	ECOG PS 0-2	ECOG PS 0-1	ECOG PS 0-2	GOG PS 0-2
Sample size	1248	1528	484	361	748
Average age (year)	60	57	61	61	60
Histology	Epithelial ovarian cancer, primary peritoneal cancer, or fallopian-tube cancer	Epithelial ovarian cancer, primary peritoneal cancer, or fallopian-tube cancer	Epithelial ovarian cancer, primary peritoneal cancer, or fallopian-tube cancer	Epithelial ovarian cancer, primary peritoneal cancer, or fallopian-tube cancer	Epithelial ovarian cancer, primary peritoneal cancer, or fallopian-tube cancer
Control arm	Cycles 1–6: C (AUC 6) + P (175 mg/m2)+ PL, q3w Cycles 7–22: PL, q3w	Cycles 1–6: C (AUC 5 or 6)+ P (175 mg/m2), q3w	Cycles 1–10: G (1,000 mg/m2 on days 1 and 8) + C (AUC 4 on day 1) + PL (15 mg/kg on day 1), q3w	Cycles 1-PD: PAC (80 mg/m2 days 1, 8, 15, and 22 q4w); or TOP (4 mg/m2, days 1, 8, 15 q4w or 1.25 mg/m2, days 1–5 q3w); or PLD (40 mg/m2 day 1 q4w)	Paclitaxel (175 mg/m2) + Carboplatin (AUC5)
Experimental arm	Cycles 1–6: C (AUC 6) + P (175 mg/m2) + Bev (15 mg/kg), q3w Cycles 7–22: Bev (15 mg/kg), q3w	Cycles 1–6: C (AUC 5 or 6)+ P (175 mg/m2)+ Bev (15 mg/kg), q3w Cycles 7–18: Bev (15 mg/kg), q3w	Cycles 1–10: G (1,000 mg/m2 on days 1 and 8) + C (AUC 4 on day 1) + Bev (15 mg/kg on day 1), q3w	Cycles 1-PD: Chemotherapy + Bev (15 mg/kg q3w or 10 mg/kg), q2w	Bev (15 mg/kg) + P (175 mg/m2) + C (AUC5), followed by Bev maintenance

PFS, progression-free survival; OS, overall survival; GOG, Gynaecological Oncology Group; ECOG, Eastern Cooperative Oncology Group; PS, performance status; C, carboplatin; AUC, area under curve; P, paclitaxel; Bev, bevacizumab; PL, Placebo; G, gemcitabine; PAC, weekly paclitaxel; TOP, topotecan; PLD, pegylated liposomal doxorubicin; PD, progressive disease.

**Table 2:** Efficacy results of 5 RCTs

References	Arms	Sample Size	Patient Characteristic	Primary Endpoint	PFS				OS			ORR (%)
					Median (months)	HR	HR, 95% CI	Median (months)		HR	HR, 95% CI	
GOG218	TC+PL	625	Newly diagnosed	PFS	10.3	0.770	0.681-0.870	39.3		0.885	0.750-1.040	NR
	TC+Bev+Bev(m)	623			14.1			39.7				NR
ICON7	TC	764	Newly diagnosed	PFS	17.5	0.930	0.830-1.050	58.6		0.990	0.850-1.140	48.0
	TC+Bev+Bev(m)	764			19.9			58.0				67.0
OCEANS	GC+PL	242	Recurrent	PFS	8.4	0.484	0.388-0.605	32.9		0.952	0.771-1.176	57.4
	GC+Bev+Bev(m)	242			12.4			33.6				78.5
AURELIA	CT(PLD or PAC or TOP)	182	Recurrent	PFS	3.4	0.480	0.380-0.600	13.3		0.850	0.660-1.080	12.6
	CT+Bev+Bev(m)	179			6.7			16.6				30.9
GOG213	TC	374	Recurrent	OS	10.4	0.614	0.522-0.722	37.3		0.827	0.683-1.005	NR
	TC+Bev+Bev(m)	374			13.8			42.2				NR

TC, Paclitaxel+Carboplatin; GC, Gemcitabine+Carboplatin; PL, placebo; Bev(m), Bevacizumab (maintenance chemotherapy); CT, PLD or PAC or TOP; PLD, pegylated liposomal doxorubicin; PAC, weekly paclitaxel; TOP, topotecan; PFS, progression-free survival; OS, overall survival; ORR, objective response rate; CI, confidence interval; NR, not reported.

### Progression-free survival

**Figure 3:**
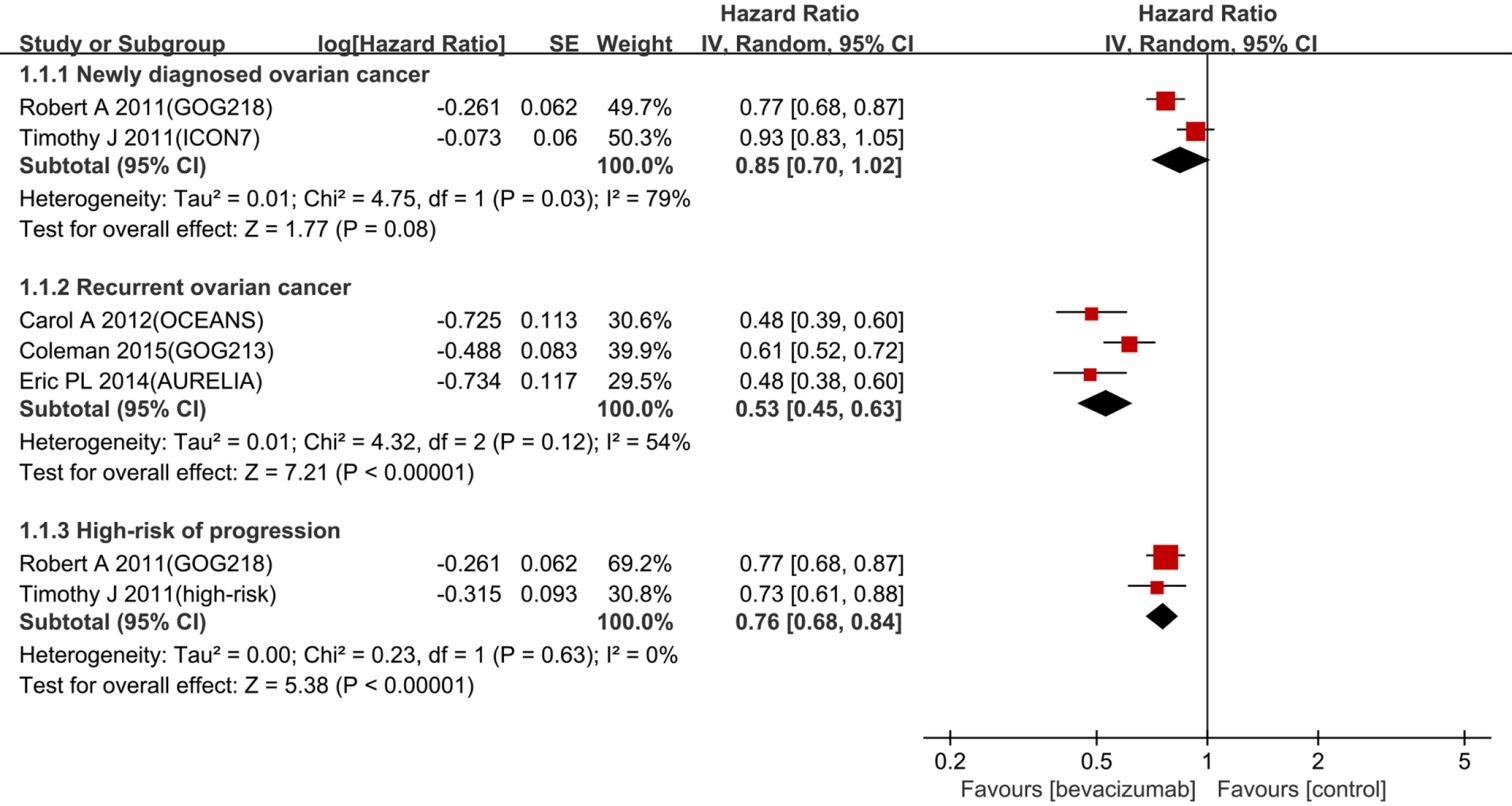
Forest plots for PFS

In the newly diagnosed setting, bevacizumab combined with chemotherapy had no statistically significant improvement in PFS (hazard ratio (HR) 0.85, 95% confidence interval (CI) 0.70-1.02, *p* = 0.03, I^2^ = 79%,) (Figure 3). In contrast, PFS was significantly improved in the recurrent setting (HR 0.53, 95% CI 0.45-0.63, *p* = 0.12, I^2^ = 54%). Considering the large heterogeneity between newly diagnosed settings (I^2^ = 79 %, *p* = 0.03), the random-effects model was utilized for further subgroup analysis. The benefit on PFS in patients with a high risk of progression was significant (HR 0.76, 95% CI 0.68-0.84, I^2^ = 0%) (Figure 3).

### Overall survival

**Figure 4:**
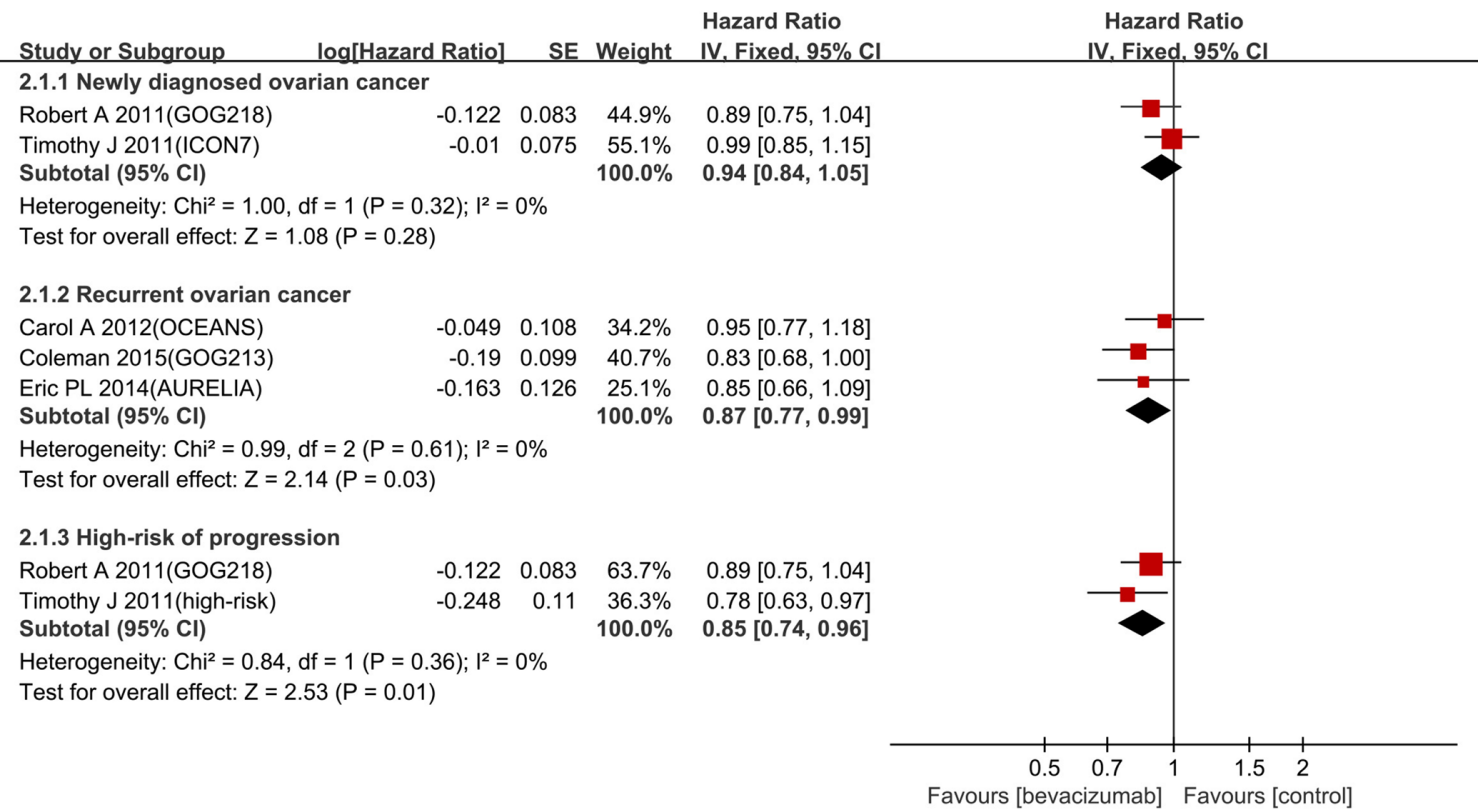
Forest plot for OS

Bevacizumab had a significantly better OS in both the recurrent setting (HR 0.87, 95% CI 0.77-0.99, I^2^ = 0%) and the patients with a high-risk of progression (HR 0.85, 95% CI 0.74-0.96, I^2^ = 0%) (Figure 4). No statistically significant improvement was identified in newly diagnosed setting (HR 0.94, 95% CI 0.84-1.05, I^2^ = 0%).

### Objective response rate

**Figure 5:**
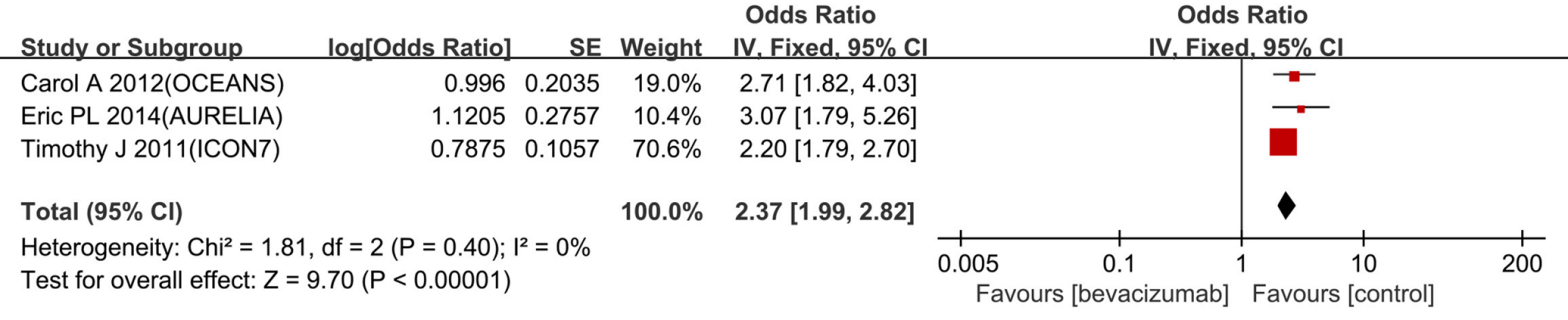
Forest plots for ORR

Three trials of objective response rate (ORR) have been reported, and the pooled odds ratio (OR) for ORR was 2.37 (95% CI 1.99-2.82, I^2^ = 0%) (Figure 5). This finding demonstrates that bevacizumab combined with chemotherapy improved the ORR more effectively compared with chemotherapy alone.

### Adverse events

**Figure 6:**
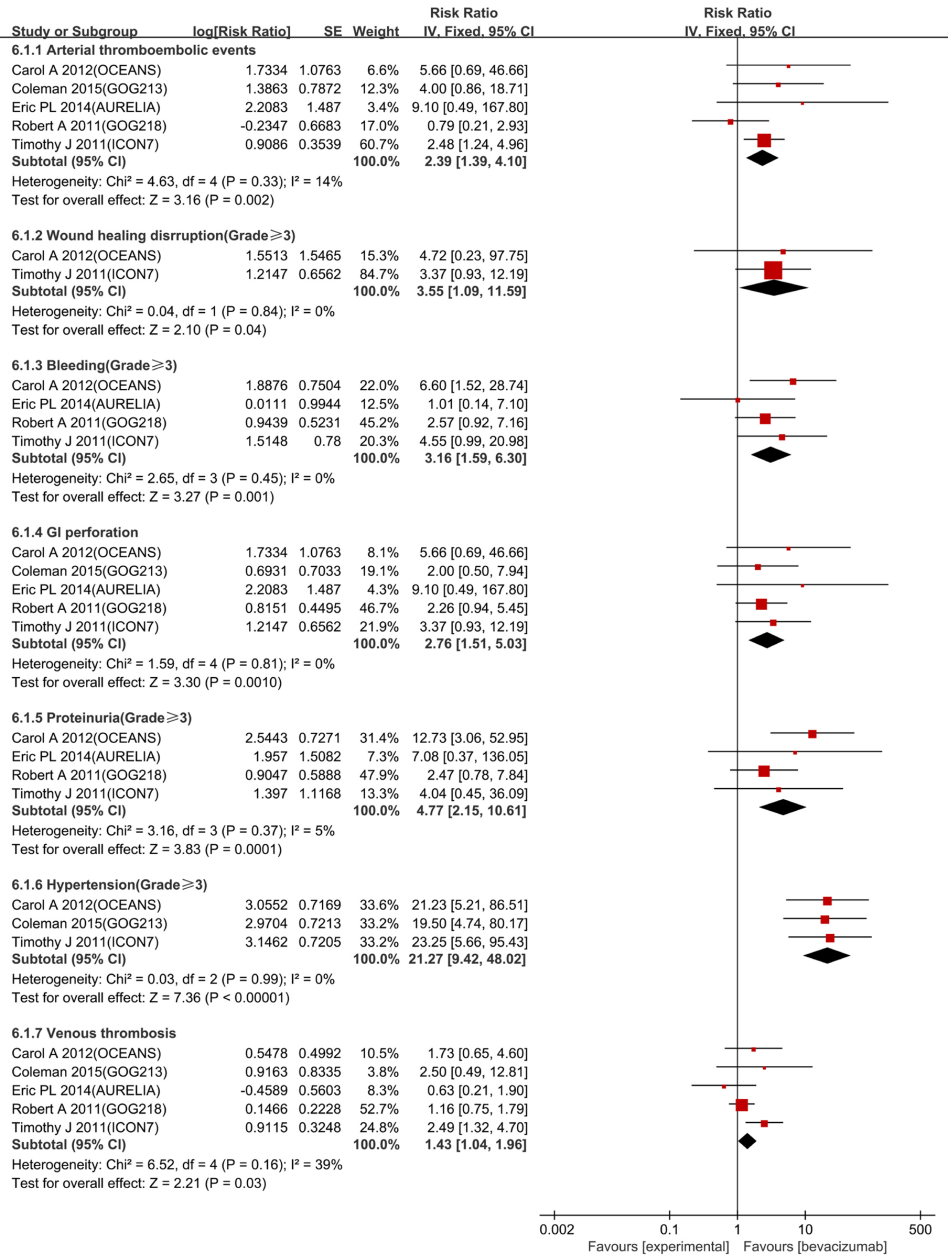
Forest plot for common adverse events

Figure 5 presents 6 common adverse events that are potentially associated with bevacizumab. Among this updated analysis, the risks of hypertension, proteinuria, bleeding, wound healing disruption, GI perforations, arterial thrombosis events and venous thrombosis events were significantly increased as follows: hypertension (risk ratio (RR) 21.27, 95% CI 9.42-48.02, I^2^ = 0%), proteinuria (RR 4.77, 95% CI 2.15-10.61, I^2^ = 0%), wound healing disruption (RR 3.55, 95% CI 1.09-11.59, I^2^ = 0%), bleeding (RR 3.16, 95% CI 1.59-6.30, I^2^ = 0%), GI perforations (RR 2.76, 95% CI 1.51-5.03, I^2^ = 0%), arterial thrombosis events (RR 2.39, 95% CI 1.39-4.10, I^2^ = 14%), and venous thrombosis events (RR 1.43, 95% CI 1.04-1.96, I^2^ = 39%) (Figure 6).

### Publication bias

**Figure 7:**
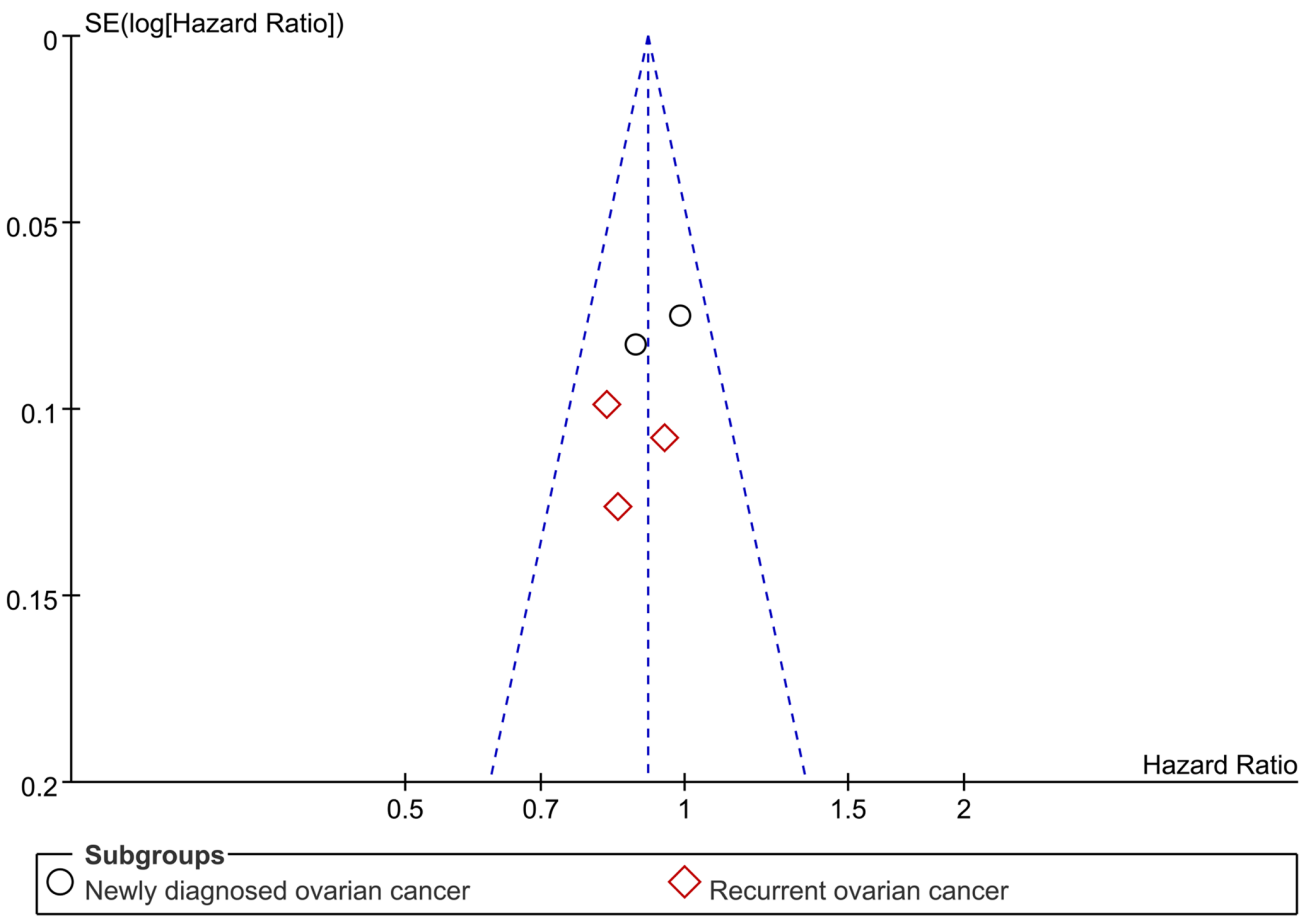
Funnel plot

Publication bias of the included trials was evaluated by funnel plot. The funnel plot for OS revealed almost symmetry, thus indicating no significant publication bias for OS (Figure 7). PFS didn't do funnel plot, because the significant heterogeneity will lead to asymmetric funnel plot. Importantly, due to the number of trials is small (< 10), funnel plots have limited power to detect publication bias [11].

## DISCUSSION

This updated meta-analysis was derived from a new RCT and final data to reassess the efficacy and safety of bevacizumab combined with chemotherapy in ovarian cancer. The conclusion is different from the previous meta-analysis. The updated results indicated that bevacizumab combined with chemotherapy significantly improved PFS and OS in patients with a high risk of progression and recurrent ovarian cancer. In addition, the ORR was significantly increased in the overall population.

Four trials (ICON7, GOG-218, OCEANS and AURELIA) were designed to observe the PFS as the primary endpoint. For recurrent ovarian cancer, bevacizumab combined with chemotherapy reduced the HR of progression by 47% compared with chemotherapy alone. For patients with a high risk of progression, the addition of bevacizumab reduced the HR of progression by 24% compared with chemotherapy alone. Notably, the pooled HR of PFS for the newly diagnosed setting was 0.85 (95%CI 0.70-1.02, I^2^ = 79%) with large heterogeneity. According to our analysis, the heterogeneity was mainly derived from the difference FIGO stages of the recruited patients. Thus, we performed further analysis in patients with a high risk of progression who were predefined in the ICON7 trial and matched all the recruited patients in the GOG-218 trial. The results indicated that the PFS was significantly improved in this subgroup (HR 0.76, 95% CI 0.68-0.84, I^2^ = 0%). Moreover, for the non-high-risk patients, bevacizumab did not improve OS (HR 1.14, 95% CI 0.93-1.40) or PFS (HR 1.03, 95% CI 0.88-1.21). This finding implies that the benefit from bevacizumab is associated with prognostic factors [9].

To our knowledge, the GOG-213 was the first phase III randomized trial designed to detect OS as the primary endpoint. Bevacizumab combined with chemotherapy reduced the HR of death by 13% in the recurrent setting and 15% in the patients with high-risk progression. The result from this updated data is consistent with a similar meta-analysis [12]. However, the OS as a secondary endpoint, the multiple lines of post-progression treatment and the crossover effect made it difficult to detect the OS benefit associated with bevacizumab in the others trials.

With the addition of bevacizumab, no significant decline in quality of life was noted even though the risk of several adverse events, such as hypertension, wound healing disruption, proteinuria, bleeding, GI perforations and thrombosis events, were relatively increased [13]. It is necessary to monitor and manage these adverse events during the bevacizumab therapy to minimize the risks [14]. If severe adverse events such as GI perforations can be controlled, bevacizumab can be used safely.

In newly diagnosed ovarian cancer, the overall population had no statistical survival benefit according to the two trials, ICON7 and GOG-218. Remarkably, in patients with a high risk of progression, the evidence implies that bevacizumab confers a survival benefit. The addition of bevacizumab to first-line treatment in ovarian cancer would be a good option for patients with poor prognoses, such as stage III or IV patients after debulking surgery. However, the survival benefit of bevacizumab in high-risk patients was concluded from subgroup analysis. The results from subgroup analyses should be noted because the consistency of patient characteristics and principle of randomization were not ensured. The evidence must be verified by another trial with a placebo control. Fortunately, there were several options for adjuvant therapy of newly diagnosed ovarian cancer. Firstly, chemotherapy based on weekly paclitaxel and carboplatin is an option. The JGOG 3016 trial [15] showed that both PFS and OS were significantly extended with the use of weekly paclitaxel and carboplatin compared with standard chemotherapy, whereas the GOG-262 trial [16] and the MITO-7 trial [17] showed that a dose-dense paclitaxel regimen did not prolong PFS significantly. The ICON8 trial (ISRCTN10356387), which addresses the same issue, is ongoing. Secondly, neoadjuvant chemotherapy accompanied with cytoreductive surgery or intraperitoneal chemotherapy could be considered in treatment [8]. Therefore, clinicians must select an appropriate front-line therapy for patients with advanced ovarian cancer.

Even if a pathological complete response is achieved, the recurrence rates are greater than 40%. Thus, almost all patients will eventually die from recurrence [18]. The most important goal in the treatment of recurrent ovarian cancer is prolonging the survival time using both an effective and well-tolerated strategy. The results of three trials (OCEANS, AURELIA and GOG-213) in this meta-analysis demonstrate that bevacizumab combined with chemotherapy is a great treatment option for recurrent ovarian cancer. Among the three trials, OCEANS and GOG-213 were aimed at platinum-sensitive ovarian cancer and AURELIA was aimed at platinum-resistant ovarian cancer relatively. The median PFS and OS of platinum-resistant patients were significantly reduced compared with platinum-sensitive patients [Table 2]. The result of a similar trial, TRINOVA-1, which was designed for platinum-resistant ovarian cancer, shows that trebananib also significantly increases the median PFS (7.2 months *versus* 5.4 months) relative to placebo [19].

Several problems must be addressed for bevacizumab-containing therapy in ovarian cancer. Firstly, the optimum dose of bevacizumab in the treatment of ovarian cancer is undefined [20]. The ICON7 trial used 7.5 mg/kg bevacizumab based on the dose in colorectal cancer, whereas the others trials used 15 mg/kg bevacizumab based on the dose for non-small-cell lung cancer. To date, no head-to-head trial has indicated the difference of survival benefit between two doses. Secondly, the efficacy of bevacizumab in recurrent patients who received bevacizumab in front-line therapy is unclear. In the TANIA trial for breast cancer and the ML18147 trial for colorectal cancer, the results indicate that bevacizumab provides clinical benefit for patients with recurrent disease who responded to first-line bevacizumab with chemotherapy [21, 22]. The MITO16 trial (NCT01802749), which addresses a similar issue in ovarian cancer, is ongoing. In addition, cost-effectiveness is essential and should be considered for the patients.

This updated meta-analysis included 5 RCTs with 4994 patients, whereas the previous publication contained 4 RCTs with 4246 patients. One additional trial, GOG-213, had final results published in abstract form from conference proceedings. Moreover, the final data from the OCEANS trial (OS) and the ICON7 trial (PFS, OS) were published recently. Previously reported preliminary data could not accurately reflect the survival benefit. The conclusion of the most recent meta-analysis showed that bevacizumab combined with chemotherapy improved PFS and OS in the front-line setting in ovarian cancer instead of improving the OS in the recurrent setting [10]. However, the result of this updated meta-analysis indicates that bevacizumab combined with chemotherapy improves PFS and OS in a recurrent setting, with no statistically significant improvement in PFS and OS for newly diagnosed OC. This result has considerable clinical significance indicating that the benefit of bevacizumab in ovarian cancer may be associated with prognosis factors, identifying patients who benefit most from bevacizumab and providing a high level of evidence for the rational use of bevacizumab in ovarian cancer.

The main limitation of this meta-analysis is the clinical heterogeneity across the studies, including the different tumor stages, the length of follow-up, the dose of bevacizumab, and the chemotherapy regimens. Secondly, we pooled HR for time-to-event data rather than assessing individual patient data. Thirdly, some negative trial results may not be published, thus excluding unpublished trials may introduce bias. Finally, this meta-analysis only included 5 RCTs, which was insufficient to analyze sensitivity.

## MATERIALS AND METHODS

### Literature search and inclusion criteria

The literature search focused on randomized controlled trials published from database inception to May 2016. Studies comparing bevacizumab plus chemotherapy with chemotherapy alone were eligible for inclusion. We searched the PubMed, EMBASE, Web of Science and Central (Cochrane clinical trials database) databases, and we also searched clinicaltrial.gov. We used the search terms “bevacizumab”, “Avastin”, “chemotherapy”, and “ovarian cancer” in various combinations. In addition, only phase III randomized trials were restricted in the search strategy. The language of an article published was not restricted.

In order to explore the general efficacy of bevacizumab and to avoid biased conclusions, we used the same criteria as the previous meta-analysis [10]. Selection criteria followed the “PICOS” principle (P, population: women with ovarian cancer; I, intervention: chemotherapy plus bevacizumab; C, comparison: chemotherapy alone; O, outcome: efficacy and safety; and S, study: randomized controlled trial). Initially, articles that did not conform to the principles were excluded by reading the title and abstract. Subsequently, full-text evaluation was adopted when the first step could not determine inclusion or exclusion, and then the irrelevant article was excluded. Two investigators independently completed all of the processes. Discrepancies were resolved by discussion.

### Quality assessment and data extraction

The risk of bias was assessed using the Cochrane Collaboration's tool. The risk of bias assessment was judged by three categories for each study: low risk (+), unclear risk (?) or high risk (-) of bias [23]. Data from each trial were classified into three domains: participant characteristics, study interventions, and outcomes. Participant characteristics contain participant sample size and stratification factors. Study interventions contain patient allocation and treatment regimens. The data of outcomes were extracted as follows: PFS, OS, ORR and incidence of adverse events. When two intervention groups were designed in the trial, only one intervention group that is most similar to the others trials was selected [24]. All processes were completed independently by two reviewers, and disagreements were resolved by discussion to reach a consensus according to Kappa index [25].

### Data analysis and statistical methods

As a time-to-event outcome, PFS and OS were evaluated by using the HR. The HR and two-sided 95% CI were extracted directly from the trial reports. The Chi-squared test and Cochran Q-test were used to evaluate heterogeneity among trials, and I^2^ > 75% indicated considerable heterogeneity [26]. We pooled PFS in a random effects model based on the large heterogeneity among the different trials. For OS, ORR and adverse events, we used the fixed effect model. We pooled the RR for the adverse events and OR for ORR to assess the efficacy and safety. Subgroup analyses were adopted to determine whether there is survival benefit for patients in the subgroup classified by prognostic factors. A high risk of progression was defined in the ICON7 trial as FIGO stage III or IV disease after debulking surgery. The high-risk progression group consisted of 502 patients and matched all populations in the GOG-218 trial. The meta-analysis software RevMan 5.3 provided by the Cochrane library was used for the data analysis. Due to the small quantity of included trials (< 10), we did not examine potential publication bias with Begg and Egger tests.

## CONCLUSIONS

This updated meta-analysis indicates that bevacizumab combined with chemotherapy significantly improved PFS and OS in both patients with high-risk of progression and patients with recurrent OC, with an increased incidence of common adverse events. However, no statistically significant survival benefit was identified in the front-line settings. ORR is improved in overall population by the addition of bevacizumab.
